# Changes, and the Relevance Thereof, in Mitochondrial Morphology during Differentiation into Endothelial Cells

**DOI:** 10.1371/journal.pone.0161015

**Published:** 2016-08-12

**Authors:** Ji Won Shin, So Hee Park, Yun Gyeong Kang, Yanru Wu, Hyun Ju Choi, Jung-Woog Shin

**Affiliations:** 1 Department of Biomedical Engineering, Inje University, Gimhae, Gyeongnam, Republic of Korea; 2 Department of Health Science and Technology, Inje University, Gimhae, Gyeongnam, Republic of Korea; 3 Research and Development Team, Gimhae Biomedical Center, Gimhae, Gyeongnam, Republic of Korea; 4 Cardiovascular and Metabolic Disease Center/Institute of Aged Life Redesign/UHARC, Inje University, Gimhae, Gyeongnam, Republic of Korea; Michigan Technological University, UNITED STATES

## Abstract

The roles of mitochondria in various physiological functions of vascular endothelial cells have been investigated extensively. Morphological studies in relation to physiological functions have been performed. However, there have been few reports of morphological investigations related to stem cell differentiation. This was the first morphological study of mitochondria in relation to endothelial differentiation and focused on quantitative analysis of changes in mitochondrial morphology, number, area, and length during differentiation of human mesenchymal stem cells (hMSCs) into endothelial-like cells. To induce differentiation, we engaged vascular endothelial growth factors and flow-induced shear stress. Cells were classified according to the expression of von Willebrand factor as hMSCs, differentiating cells, and almost fully differentiated cells. Based on imaging analysis, we investigated changes in mitochondrial number, area, and length. In addition, mitochondrial networks were quantified on a single-mitochondrion basis by introducing a branch form factor. The data indicated that the mitochondrial number, area per cell, and length were decreased with differentiation. The mitochondrial morphology became simpler with progression of differentiation. These findings could be explained in view of energy level during differentiation; a higher level of energy is needed during differentiation, with larger numbers of mitochondria with branches. Application of this method to differentiation into other lineages will explain the energy levels required to control stem cell differentiation.

## Introduction

Mitochondria, the major energy producers in the cell, are known to be involved in various cellular activities and/or functions, including proliferation, aging, and apoptosis [1–2]. Recent studies of mitochondrial morphology have attracted a great deal of attention as morphological changes have been shown to be closely related to their functions and roles, which are thought to affect other cellular activities [3]. For example, it was reported that the upregulation of cyclin E is accompanied with mitochondrial hyperfusion in cells during the G1–S phase transition and changes in mitochondrial biogenesis [4–5]. In addition, changes in mitochondrial morphology were shown to accompany oxidative phosphorylation activities associated with glucose or galactose [6]. Thus, mitochondrial morphology changes continuously accompanying various specific cellular functions. It is also widely known that mitochondria play important roles in the proliferation, differentiation, and maintenance of the stemness of stem cells [4–9]. For example, Ishihara et al. reported increases in Drp1 expression on differentiation of embryonic stem cells into neuronal cells [7], and De Palma et al. reported decreases in Drp1 expression during the myogenic differentiation of embryonic stem cells. With regard to morphological changes in relation to stem cell research, most previous reports mentioned only fragmentation or elongation; i.e., the changes were discussed in a qualitative manner. Chung et al. reported elongation of mitochondria during cardiomyogenic differentiation of embryonic stem cells along with increases in OPA1 and MFN1 expression and decreases in Dnm1 expression [10].

Although mitochondria in vascular endothelial cells do not occupy a larger volume compared with other closely related cell types and play important roles in cellular processes—such as biogenesis, cellular dynamics, mitophagy, ROS production, and calcium homeostasis [11–15]—there have been no previous studies of mitochondrial morphological changes during endothelial differentiation. Therefore, this study was performed to quantitatively investigate the morphological changes of mitochondria during differentiation of mesenchymal stem cells into vascular endothelial cells utilizing digital image-processing techniques. Specifically, we acquired images of mitochondria from single cells and examined mitochondrial morphology depending on the stage of differentiation; i.e., undifferentiated stem cells, differentiating cells, and almost fully differentiated cells. We acquired images of mitochondria to allow meaningful statistical analyses: 90 images on average, more than 30 images at least for each stage. Finally, we attempted to explain morphological changes at each stage during differentiation from the viewpoint of energy requirements.

## Materials and Methods

### Cell culture and induction of endothelial differentiation

Human mesenchymal stem cells (hMSCs) were purchased from Lonza (Walkersville, MD, USA). The cells were cultured according to the manufacturer’s protocol up to passage #4 and seeded at 1×10^4^ cells/cm^2^ on fibronectin-coated cover glasses on a miniature fluid-chip and cultured for 24 hours to allow stabilization. Endothelial differentiation of hMSCs was induced in Dulbecco’s modified Eagle’s medium with low glucose (Gibco, Grand Island, NY, USA) containing vascular endothelial growth factor (VEGF, 50 ng/mL; PeproTech, Rocky Hill, NJ, USA), 5% fetal bovine serum, 100 U/mL penicillin, and 100 mg/mL streptomycin. Shear stress was applied as described below for 24 hours after stabilization.

### Flow-induced shear stress

**Fig 1:**
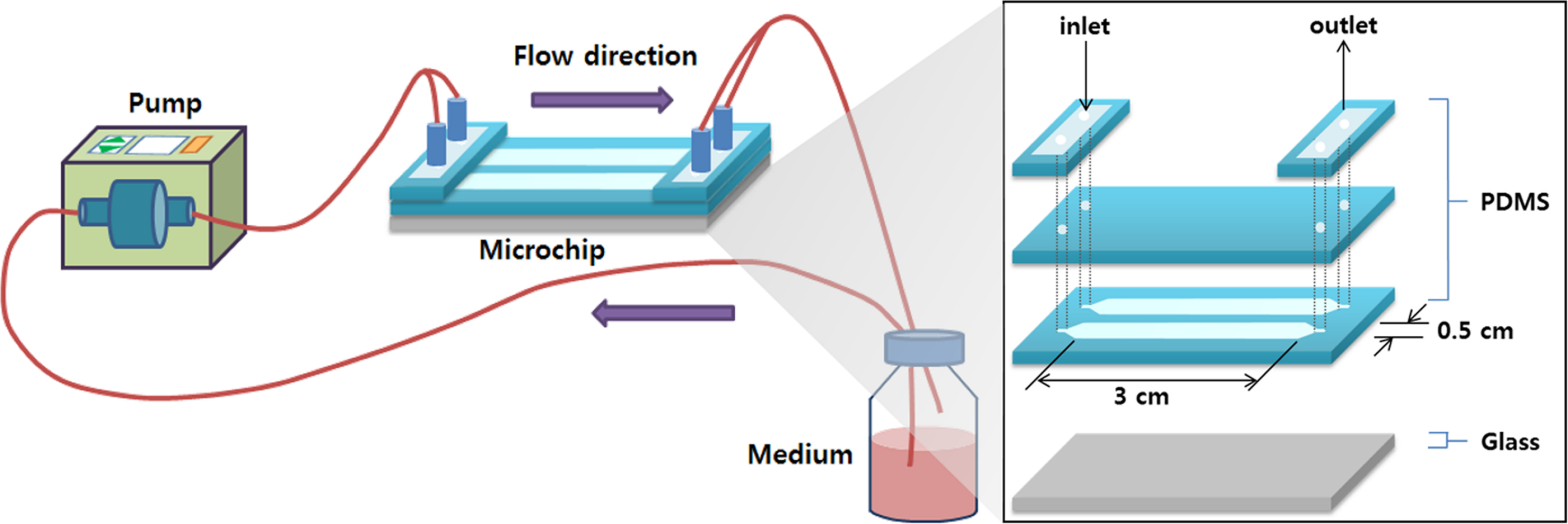
Flow system for inducing shear stress.

Fig 1 shows a schematic representation of the flow-induced shear stress system. A gear pump was used to provide a steady flow into the system. The body of a miniature fluid chip was fabricated utilizing a commercially available kit (Sylgard 184 Silicone Elastomer Kit; Dow Corning Corp., Midland, MI, USA). The mixture of polydimethylsiloxane (PDMS) and hardening agent (1:10) was poured into a mold and incubated at 70°C for 2 hours to form two parallel channels. The type of flow was confirmed to be a fully developed laminar flow (Reynolds number < 2100). By adjusting the flow rate, two magnitudes of shear stress (2.5 or 10.0 dyne/cm^2^) were applied for 24 hours after stabilization based on the formula:

$$\tau =6\mu Q/bh^{2},$$

where τ is the wall shear stress, μ is the viscosity (0.001 Pa·s), Q is the fluid flow rate (0.125 or 0.5 mL/min), b is the width (0.5 cm) of the flow channel, and h is the height (0.01 cm) of the flow channel.

### Mitochondrial staining and immunocytochemistry

Mitochondria were stained with 200 nM MitoTracker Deep Red FM (Invitrogen, Carlsbad, CA, USA) at 37°C for 30 minutes. After staining, the cells were fixed with 4% paraformaldehyde and treated cold acetone to retain mitochondrial staining according to the manufacturer’s guideline. Then, anti-von Willebrand factor (vWF) antibody (1:200; Santa Cruz Biotechnology, Santa Cruz, CA, USA) and Alexa Fluor 488 (1:200 dilution; Invitrogen) were used for immunocytochemistry to confirm endothelial differentiation. Also, nucleus was visualized with 0.5ug/ml of Hoechst 33358 (Invitrogen). Mitochondrial networking and endothelial differentiation were examined by confocal laser scanning microscopy (×400, LSM 510; Carl Zeiss, Oberkochen, Germany).

### Quantification of mitochondrial morphological changes

The number of cells selected per group was 90 on average. The measurement/calculation of the number and area of mitochondria within a cell was performed based on binary images through the thresholding process. The average mitochondrial length was calculated by skeletonizing from the binary images and normalized to the number of mitochondria. MATLAB 2012a (MathWorks, Natick, MA, USA) was used to analyze images of single cells acquired within the region of interest. Specifically, the functions of im2bw and bwmorph were used for binarization and skeletonization, respectively.

**Fig 2:**
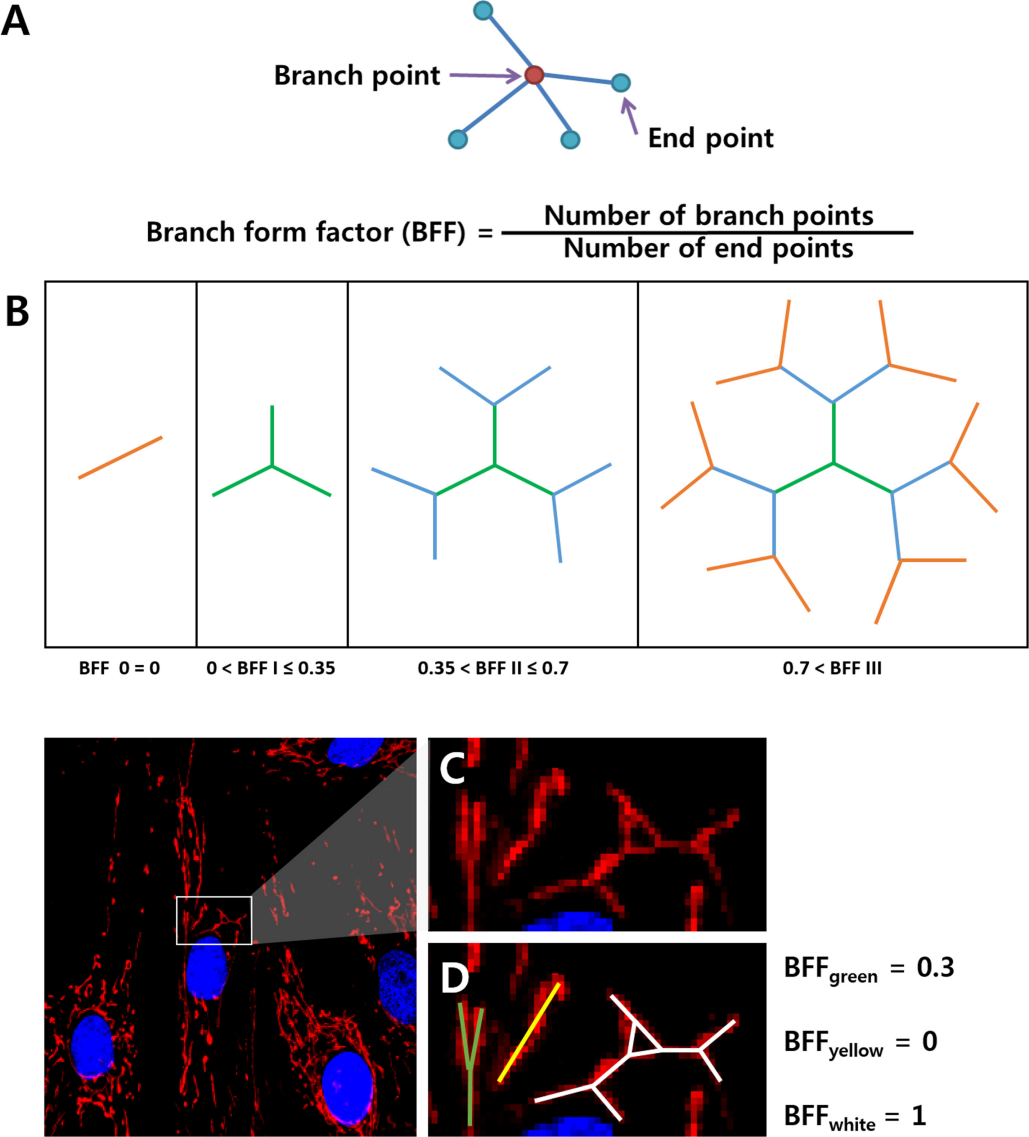
Branch form factor (A) Definition of branch form factor (B) Classification of branch form factor with complexity (C) Mitochondrial morphology in a cell (D) Sample calculation of BFF after skeletonization.

To quantify the networking of a mitochondrion, we defined a branch form factor (BFF) as shown in Fig 2(A). Three conveniently classified typical morphologies are shown based on the range of BFF adopted in this study (Fig 2(B)). The BFF was calculated by counting the number of branch points and end points within a mitochondrion. A branch point was considered as when more than three end points exited a mitochondrion; i.e., the BFF was considered to be zero when the mitochondrion had fewer than three end points, such as those with a rod shape. Fig 2(C) and 2(D) show how BFF value was calculated based on the corresponding mitochondrial morphology.

### Statistical analysis

One-way analysis of variance (ANOVA) was performed using PASW Statistics 18 (SPSS Inc., Chicago, IL, USA). When ANOVA indicated a significant difference among groups, the difference was evaluated using the least-significant difference (LSD) test. All data are presented as means ± standard deviation (SD). In all analyses, p < 0.05 was taken to indicate statistical significance.

## Results & Discussion

### Shear stress with VEGF induces differentiation of hMSCs into endothelial-like cells

**Fig 3:**
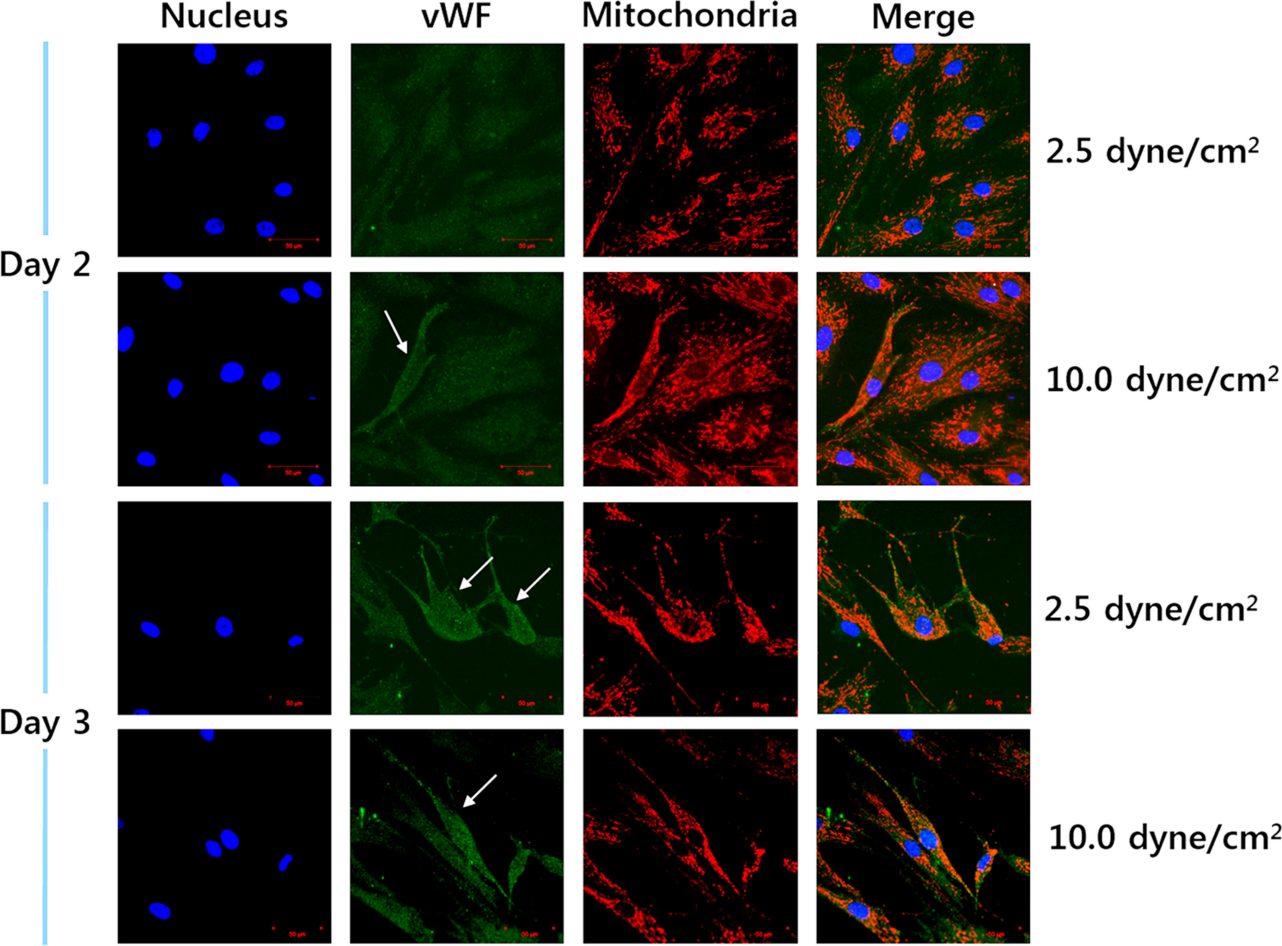
Confirmation of endothelial differentiation. White arrows indicate vWF-positive cells (Scale bar: 50 μm).

vWF is generated only by vascular endothelial cells, and it is therefore widely used as a typical endothelial differentiation marker [16]. Some hMSCs were effectively differentiated into endothelial cells when simultaneously subjected to shear stresses and VEGF (50 ng/mL) for 72 hours (Fig 3). Specifically, some of the cells began to express vWF at 24 hours (day 2) after induction with higher shear stress (10.0 dyne/cm^2^) and VEGF. This continued even up to 72 hours (day 3). However, fewer cells were found to express vWF under conditions of lower shear stress (2.5 dyne/cm^2^) with VEGF on day 2. Some of the cells under lower shear stress began to show vWF expression on day 3. No expression of vWF was detected in the group treated with VEGF alone even up to 72 hours. Other extra figures were included for more understanding. S1 Fig shows typical stained images representing vWF-positive and -negative cell along with stained mitochondria at day 7. S2 Fig indicates cells did not show vWF without VEGF while they showed vWF with VEGF at day 7 without shear stress. Other differentiation related marker (Flk-1) was also detected under higher shear stress and VEGF even at day 2 as shown in S3 Fig. These results were supported by a previous report that adipose-derived stem cells were highly differentiated into endothelial cells when exposed to shear stresses in the presence of growth factors [17–18]. Therefore, our findings indicated that the maturity of differentiation at a certain point can be ordered as follows: MSCs < group with VEGF alone < group with VEGF and lower shear stress (2.5 dyne/cm^2^) < group with VEGF and higher shear stress (10.0 dyne/cm^2^).

Specifically, vWF was expressed by larger numbers of cells when exposed to higher shear stress with VEGF. These observations based on vWF staining enabled us to distinguish differentiated from differentiating cells, and therefore we could acquire images to determine the morphological changes occurring in mitochondria during differentiation. In addition, these results reconfirmed that shear stress with VEGF can promote the differentiation of hMSCs into endothelial cells.

### Changes in mitochondrial number, area, and length within a cell during differentiation

As we cannot expect all stem cells to differentiate into the desired lineage even under the appropriate conditions for differentiation, we distinguished differentiated from undifferentiated cells by detecting a typical epithelial differentiation marker, vWF.

**Fig 4:**
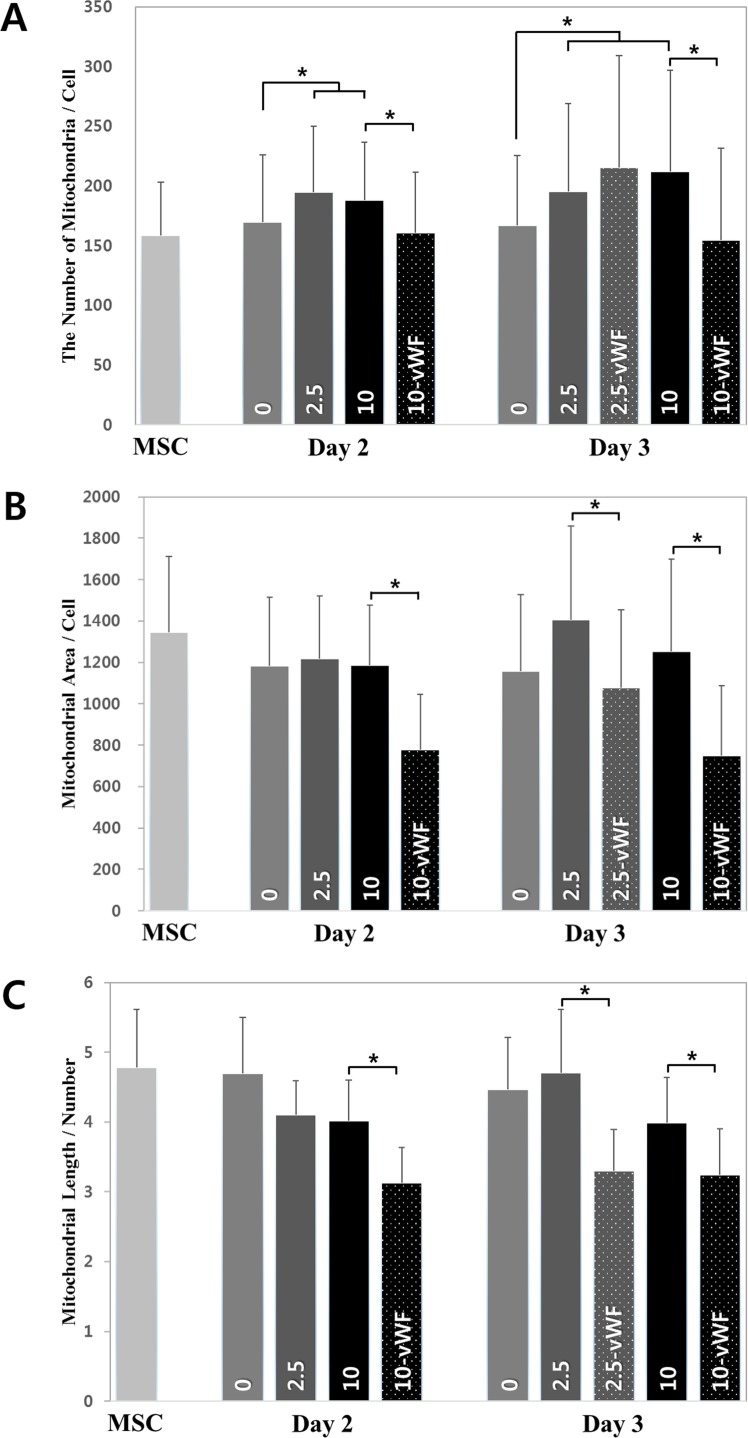
Mitochondrial morphologies during endothelial differentiation. (A) The number of mitochondria in a cell. (B) Mitochondrial area in a cell. (C) Mitochondrial length normalized relative to mitochondrial number in each cell.

The average number of mitochondria in a cell during differentiation is shown in Fig 4(A). The number of mitochondria in the cell under shear stress (2.5 dyne/cm^2^ or 10.0 dyne/cm^2^) tended to increase during the early stages of differentiation when compared with that in the cell without shear stresses. Then the number was to decrease as the cell enters the late stage of differentiation. There were fewer mitochondria in cells positive for vWF compared to those without this marker in either the undifferentiated or differentiating state. However, there was no significant difference in the number of mitochondria between groups under lower shear stress (~2.5 dyne/cm^2^) regardless of vWF expression on day 3. These observations suggested that the number of mitochondria per cell would decrease with differentiation as the higher level of shear stress (~10.0 dyne/cm^2^) accelerated differentiation compared to the lower level (~2.5 dyne/cm^2^).

The average total area of mitochondria per cell, which was calculated based on the area measured, was found to decrease with progression of differentiation in the direction of the endothelial lineage (Fig 4(B)). This result was supported by the observation that the total volume of mitochondria in a vascular endothelial cell is only ~5% of the whole cell volume, while that in cardiomyocytes is almost 30% [19–20]. Significant differences were found only between cells with and without vWF. The average length of a mitochondrion in a cell, which was calculated through the skeletonization process, showed similar trends to the total mitochondrial area in the cell (Fig 4(C)).

### Changes in mitochondrial networks during endothelial differentiation

In addition to alterations in the number, area, and length of mitochondria, it is also important to investigate changes in mitochondrial networks during differentiation. This is because energy metabolism occurs during differentiation, which is related to changes in the mitochondrial network [21]. However, most previous studies addressed these network changes in terms of fission or fusion with related proteins in a qualitative manner. However, the present study introduced the BFF for quantitative analysis of mitochondrial network changes during differentiation. As shown in Fig 2(B), the BFF value can be used to quantify the degree of network complexity of a mitochondrion, with higher and lower values corresponding to complicated and simple networks, respectively.

**Fig 5:**
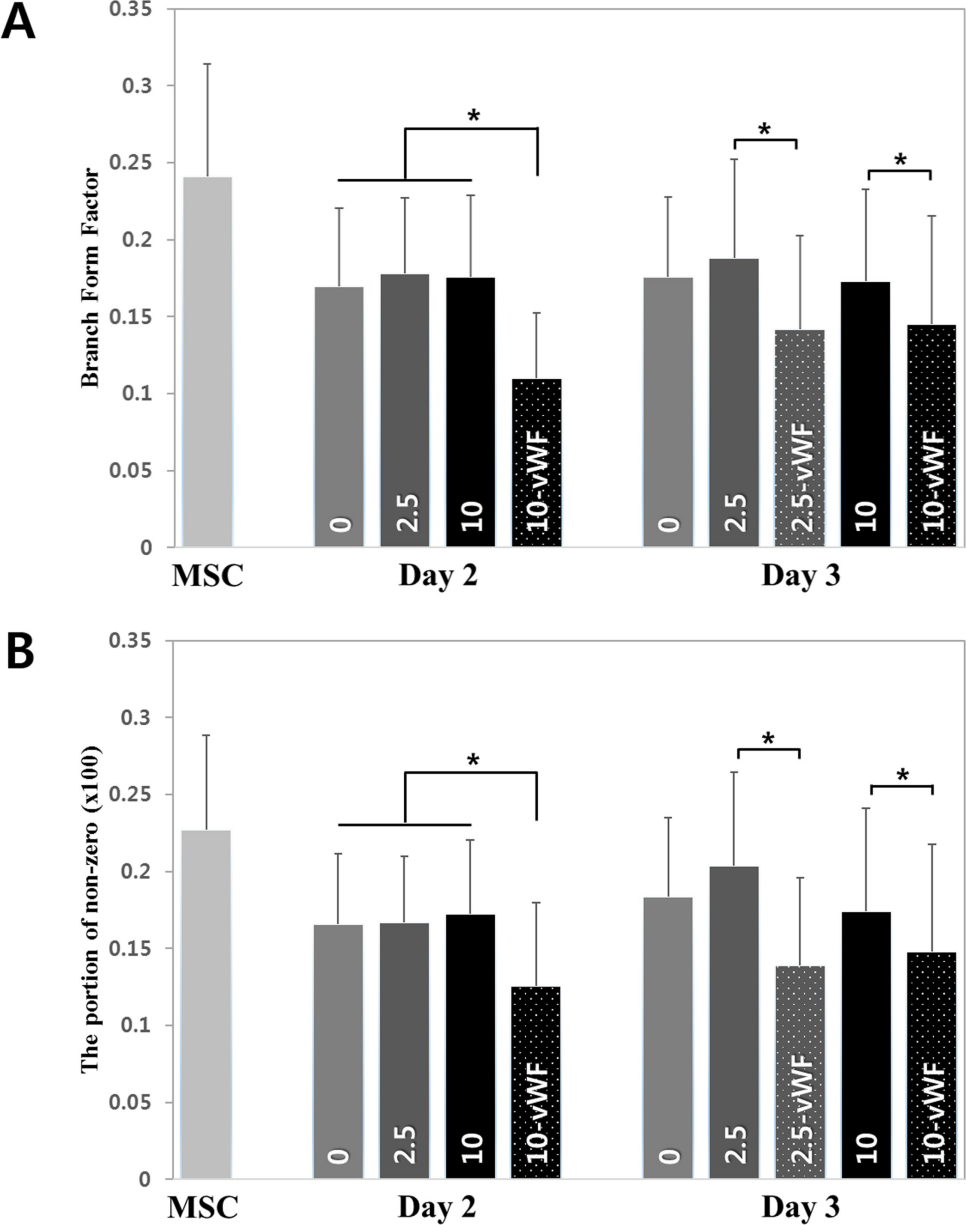
Mitochondrial network during endothelial differentiation. (A) Branch form factor to describe mitochondrial network during endothelial differentiation. (B) Percentage of non-zero branch form factor in mitochondrial morphology.

The average BFF values in each group are shown in Fig 5(A). Significantly lower values were obtained in groups expressing vWF. All values in the differentiating group were similar, but lower than those of undifferentiated MSCs. Therefore, the mitochondrial network in the cell becomes simpler with differentiation along the endothelial lineage.

**Fig 6:**
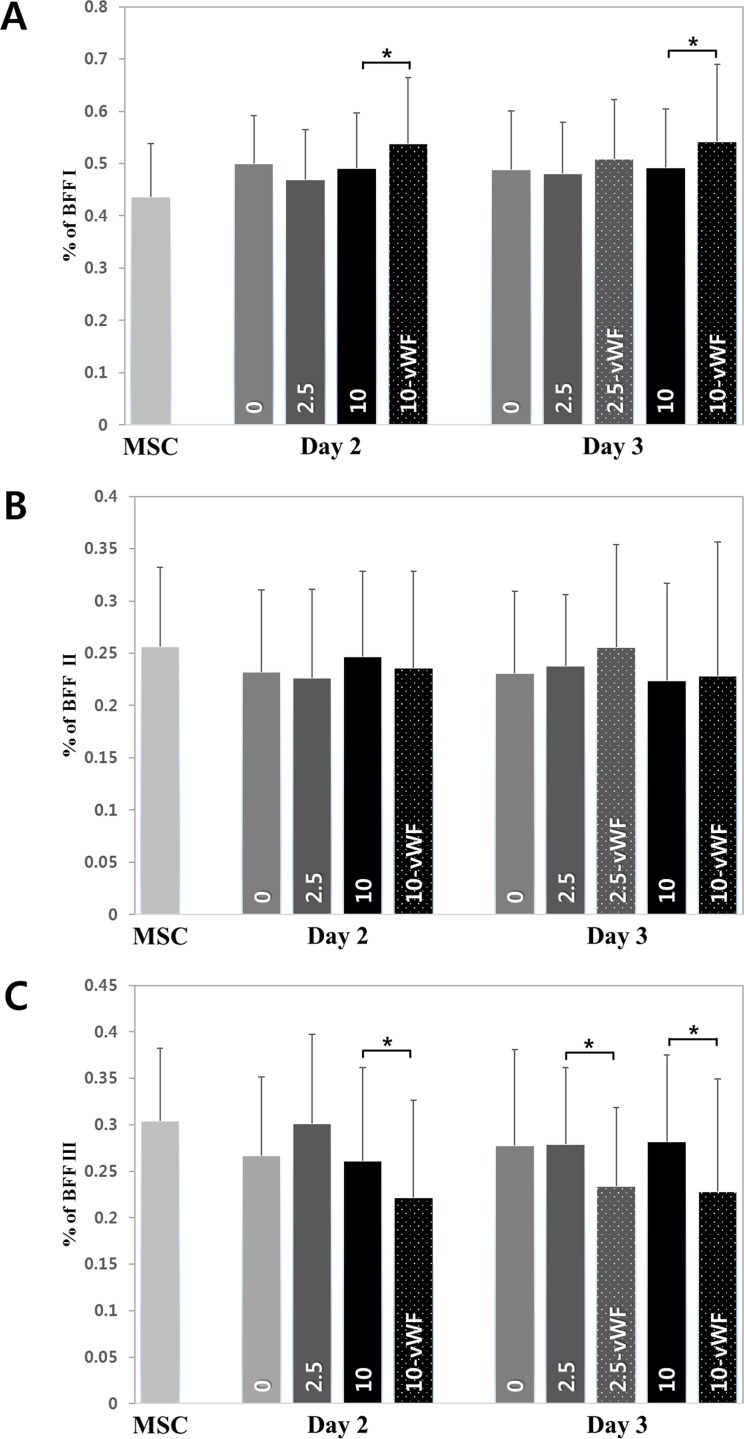
Classification of branch form factor. (A) Percentage of BFF I (0 <BFF ≤ 0.35). (B) Percentage of BFF II (0.35 < BFF ≤ 0.7). (C) Percentage of BFF III (0.7 < BFF).

The percentages of mitochondria with a non-zero BFF value in each group are shown in Fig 5(B). At most, 25% of all mitochondria per cell had a non-zero BFF value, indicating that these mitochondria have more than three end points. That is, most mitochondria have a simple shape. However, the percentages of mitochondria with a non-zero BFF value in differentiating and differentiated cells were lower than that in MSCs. Especially, the percentages of mitochondria with a non-zero BFF value in the differentiated groups (10-vWF, 2.5-vWF) were significantly lower even than those in the differentiating groups. To investigate mitochondrial network changes in greater detail, BFF values were divided into three ranges and the corresponding percentages were calculated (Fig 6). Note that each percentage was calculated based on the population excluding those with a BFF value of zero. There was no difference in the middle value range, BFF II. However, significant differences were found in the lower (BFF I) and higher (BFF III) value ranges between the vWF-expressing and non-expressing groups. In fact, Fig 6(A) and 6(C) are complementary to each other as mitochondria with a BFF value of zero were excluded from the analyses. Fig 6(A) and 6(C) again confirm that many, although not all, mitochondria have increasingly simpler shapes with progression of differentiation. When the differentiation process has almost completed; i.e., the cells are stabilized, the mitochondrial network has a significantly simpler shape than that seen in undifferentiated cells.

**Fig 7:**
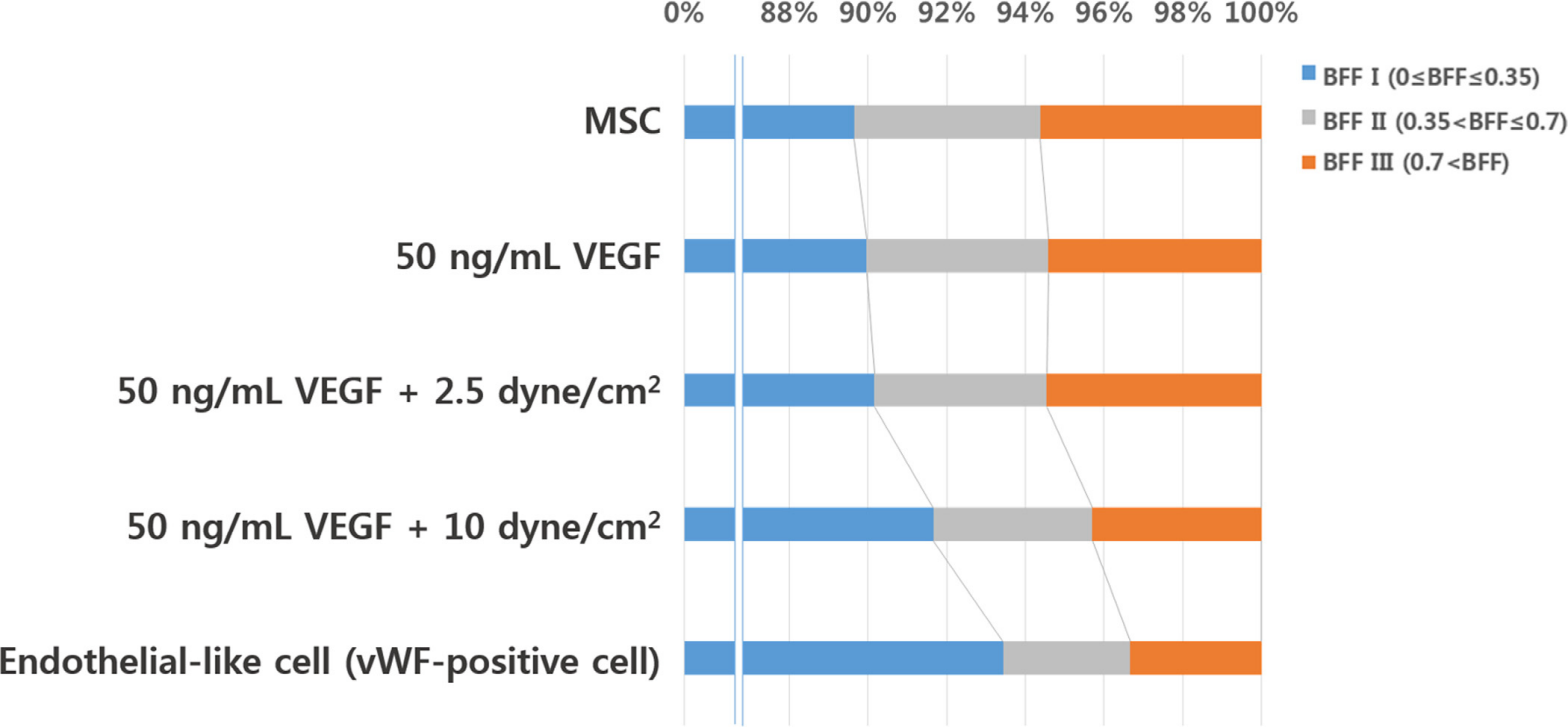
Rearranged branch form factor with endothelial differentiation regardless of culturing time.

Fig 7 shows a bar graph consisting of five categories. Each bar shows the percentage of mitochondria based on their BFF values regardless of culture duration. The first and last bars represent the status of undifferentiated (MSCs) and differentiated (vWF-expressing) cells, respectively, while the remaining three bars represent differentiating cells. Not all cells are likely to differentiate into the endothelial lineage even under the same environmental conditions. Also note that our experiments lasted only 3 days. Therefore, these five groups were reclassified into three categories regardless of culture period: MSCs, differentiating, and differentiated cells. In addition, we can figure out that cells were to be differentiated earlier when higher stress with VEGF from Fig 3. And it is obvious that cells under shear stress started differentiating regardless of shear stress magnitude as VEGF was added to all groups. Specifically, we can explain three differentiation groups more in detail as follows. When cells were under shear stress they can be assumed to be more close to express vWF than under no shear stress. Same assumption can be made between two groups: under lower and higher shear stress. Please also note that we selected almost 90 cells from each group in average to have the statistical relevance. We can easily figure out the changes in mitochondrial network that the shape was to be simpler (lower BFF value) along the differentiation.

Concrete mechanism or evidence of the relationship between mitochondria morphology and biogenesis activities has not been established so far. However, it has been observed and reported that changes in mitochondrial morphology are accompanied with various cellular activities [22–23]. Therefore, the observation of mitochondrial morphology is known to be worth to be investigated. Consequently, several models or hypothesis for mitochondrial morphology/networks and various cellular activities have been proposed. Westermann [24] proposed a hypothesis that cells have relatively higher respiratory activity when mitochondria show hyperfused network. Also, he claimed when mitochondria show fragmented shape cells demands lower energy or resting state, consequently cells show relatively lower respiratory activity. Other report found that cells have higher oxidative capacity when mitochondria show more fused [25]. Also it was reported that cells show lower oxidative capacity when many of mitochondria were shown fission state. Therefore, the basic parameters such as number, length, area, and BFF introduced in this study are surely worth to be investigated. In this study the number of mitochondria, average area of all mitochondria, and average length of mitochondria per cell decreased when the cells were almost fully differentiated. In this study the number of mitochondria, average area of all mitochondria, and average length of mitochondria per cell decreased when the cells were almost fully differentiated. In addition, the mitochondrial network showed a reduction in complexity with progression of the differentiation process. Note that the first, second, and third bars in Fig 7 indicate an almost identical distribution. However, the fourth bar showed an increased percentage of BFF I, indicating that mitochondria had become simpler in shape. This trend was also observed when vWF was expressed (fifth bar). A higher BFF value; e.g., when the mitochondrial network is in the region of BFF III, indicates that mitochondria may have several branches and consequently a large surface area. Mitochondria have a higher level of potential energy to be spent for various purposes at this stage. This increased energy level may be achieved due to biochemical (VEGF) and mechanical (shear stress) input. After this acquired energy has been spent in differentiation, initiating the differentiation mechanism, or in as-yet-unidentified pathways, their energy level decreased, and consequently the mitochondria became smaller in size and simpler in shape, as indicated by a lower BFF value.

Note that this study focused on the changes in morphology, number, area, and length of mitochondria during differentiation of MSCs into the endothelial lineage, and not any other lineage. Other cell lineages may require more energy to initiate the differentiation process. In such cases, we would expect the percentage of higher BFF values to be greater than in the differentiating groups shown in Fig 7.

## Conclusion

This study was performed to investigate various changes in mitochondria by introducing the concept of BFF along with mitochondrial number, area, and length. These results can also be used to estimate and compare potential energy levels, which would be specific to each lineage, to determine whether the cell is in the undifferentiated, differentiating, or almost fully differentiated state. With regard to engagement of mechanical stimuli for regulating stem cell differentiation, their patterns have been chosen after analyzing related genes or proteins. In this work we have shown the changes in mitochondria morphology during differentiation under mechanical stimulation along with biochemical reagents. Therefore, this can be also another parameter in selecting patterns of mechanical stimulation in promoting stem cell differentiation. In addition, this was the first study based on quantitative results regarding mitochondrial network changes during endothelial differentiation, which were explained in relation to energy level. However, this study also had several limitations. We only acquired two-dimensional images of mitochondria rather than three-dimensional images even we detected and acquired mitochondrial images as many as possible (~200 mitochondrial images/cell) for the statistical support. Also, the stipulation proposed through this study may not be applicable to differentiation to other specific lineages. Further study by accompaniment of such image-based studies with morphologically related protein studies would result in the findings regarding mitochondrial morphology and its role in relation to cellular activities, especially in differentiation, being more physiologically and biologically relevant. In addition, any other image-based analysis techniques such as fractal and textural (gray-level co-occurrence matrix; GLCM) are recommended to be adopted as potential tools in detecting cell and/or organelles morphology changes in investigating various cellular activities.

## Supporting Information

S1 Fig The stained images: cells which expressed vWF or not along with its stained mitochondria. (TIF)

S2 Fig Confirmation of differentiation with or without VEGF at day 7 without shear stress. (TIF)

S3 Fig Flk-1 expression under simultaneous engagement of shear stress and VEGF (50 ng/ml) at day 2. (TIF)
