# Supplementary figures and images for: Changes, and the Relevance Thereof, in Mitochondrial Morphology during Differentiation into Endothelial Cells
Source: PLoS One. 2016 Aug 12;11(8):e0161015. doi: 10.1371/journal.pone.0161015 (PMC4982679; doi:10.1371/journal.pone.0161015)

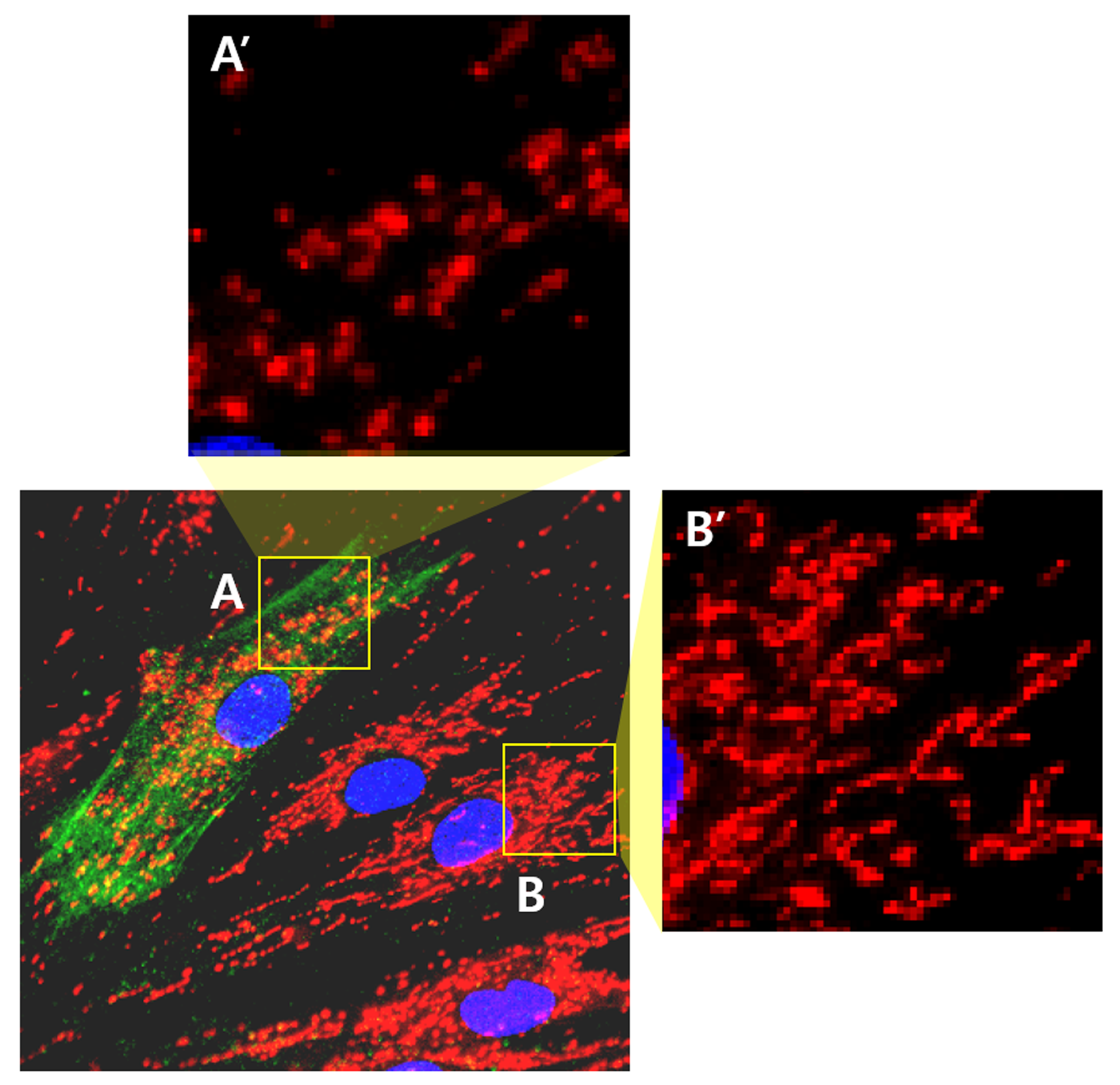

Supplement: S1 Fig — (TIF) [file pone.0161015.s001.tif]

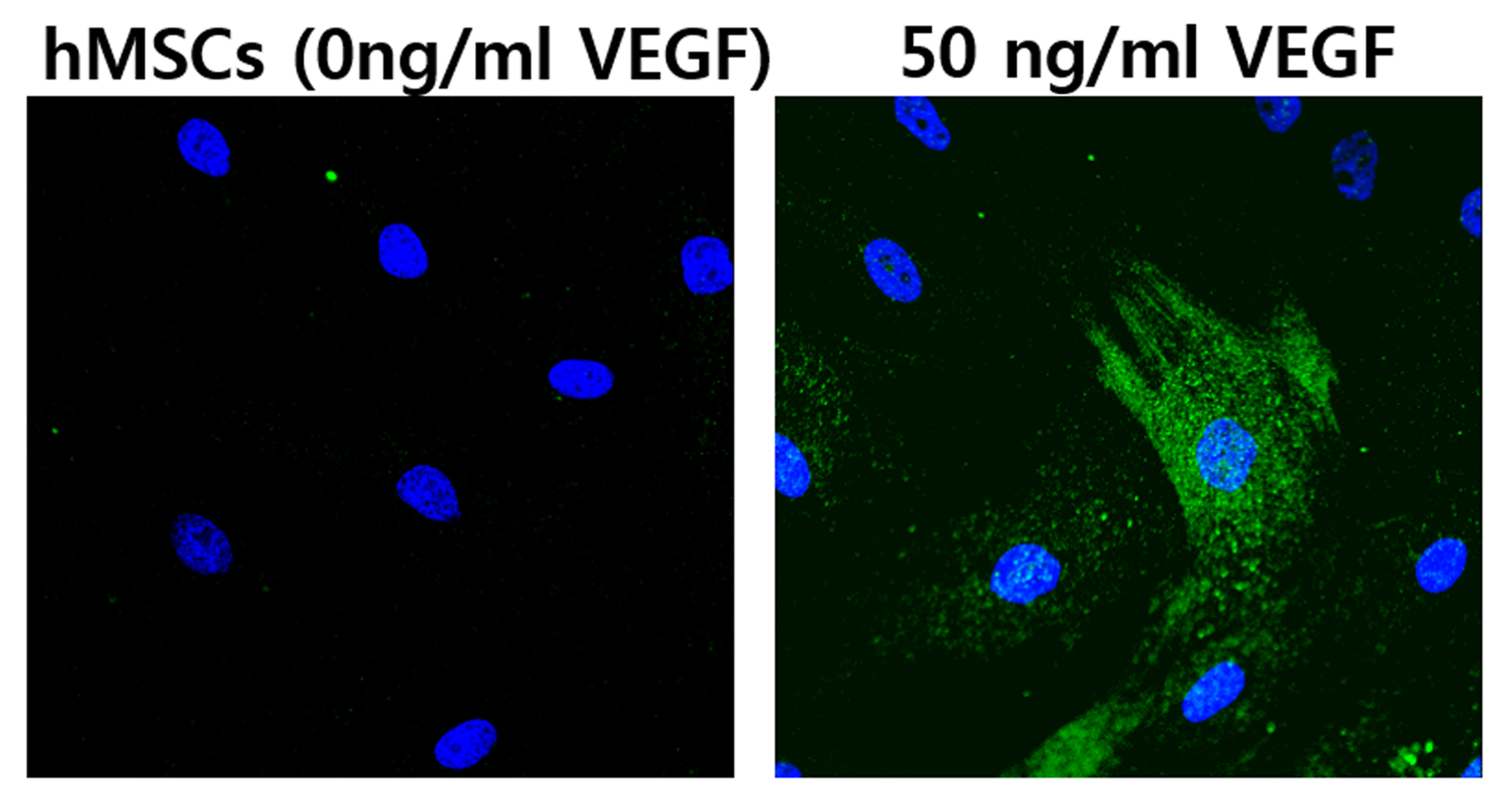

Supplement: S2 Fig — (TIF) [file pone.0161015.s002.tif]

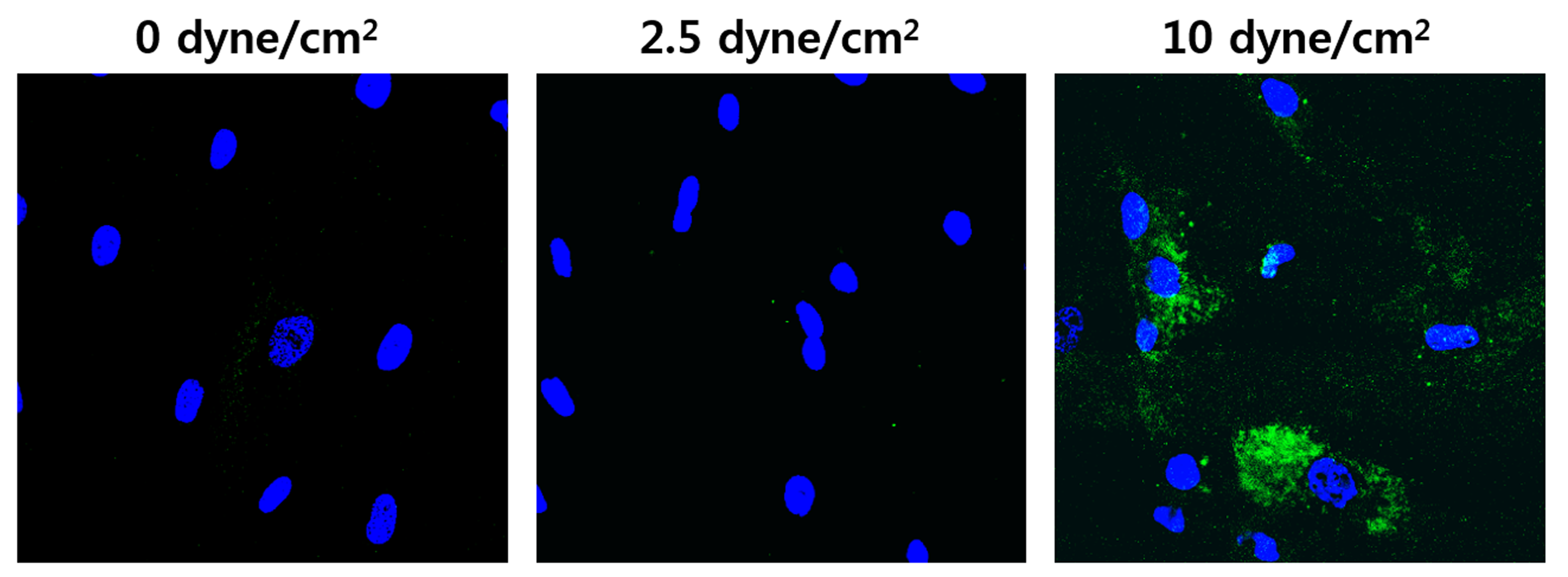

Supplement: S3 Fig — (TIF) [file pone.0161015.s003.tif]
